# Detecting the Radiative Decay Mode of the Neutron

**DOI:** 10.6028/jres.110.064

**Published:** 2005-08-01

**Authors:** B. M. Fisher, F. E. Wietfeldt, M. S. Dewey, T. R. Gentile, J. S. Nico, A. K. Thompson, K. J. Coakley, E. J. Beise, K. G. Kiriluk, J. Byrne

**Affiliations:** Tulane University, New Orleans, LA 70118 USA; National Institute of Standards and Technology, Gaithersburg, MD 20899-8461 USA; National Institute of Standards and Technology, Boulder, CO 80305-3328 USA; University of Maryland, College Park, MD 20742 USA; University of Sussex, Falmer, Brighton BN1 9RH UK

**Keywords:** beta decay, neutron decay, radiative decay, radiative corrections

## Abstract

Beta decay of the neutron into a proton, electron, and electron antineutrino is occasionally accompanied by the emission of a photon. Despite decades of detailed experimental studies of neutron beta-decay, this rare branch of a fundamental weak decay has never been observed. An experiment to study the radiative beta-decay of the neutron is currently being developed for the NG-6 fundamental physics endstation at the National Institute of Standards and Technology (NIST) Center for Neutron Research (NCNR). The experiment will make use of the existing apparatus for the NIST proton-trap lifetime experiment, which can provide substantial background reduction by providing an electron-proton coincidence trigger. Tests and design of a detector for gamma-rays in the 10 keV to 200 keV range are under development. The need for a large solid-angle gamma-ray detector that can operate in a strong magnetic field and at low temperature has led us to consider scintillating crystals in conjunction with avalanche photodiodes. The motivation and experimental technique will be discussed.

## 1. Radiative Beta-Decay of the Neutron

Like all elementary particle decays to charged particles in the final state, the beta decay of the free neutron has a radiative mode: $n\to p+e^{-}+{\bar{\nu }}_{e}+\gamma$. Despite decades of detailed experimental studies of neutron beta-decay (including very precise measurements of the neutron lifetime and beta-decay correlation coefficients), this rare decay branch has never been observed, while it has been extensively investigated in more exotic systems [1].

**Fig. 1:**
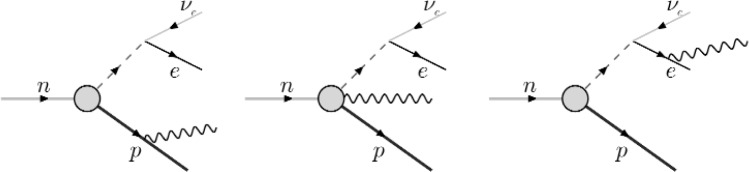
Contributions to radiative neutron decay, showing (from left to right) proton bremsstrahlung, radiation directly from the weak vertex, and electron bremsstrahlung.

**Fig. 2:**
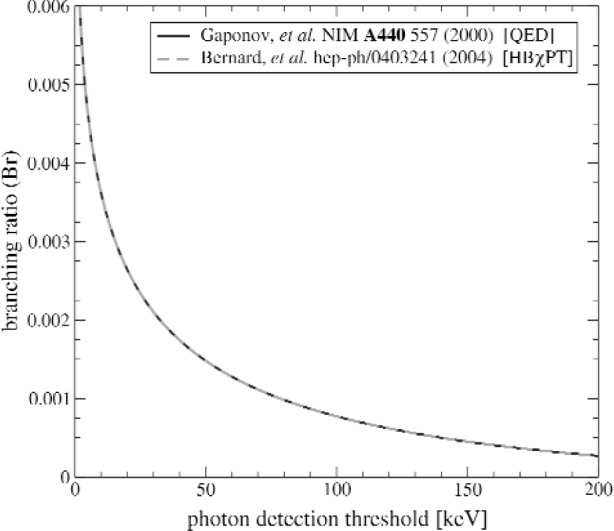
Calculations of the branching ratio for radiative neutron beta-decay. Shown are the calculations of Ref. [2] (solid line), and the HBχPT calculation (dashed line) of [3].

Recently, theoretical investigations of this process have been underway. Gaponov and Khafizov calculated the photon energy spectrum and branching ratio within a quantum electrodynamics framework [2], while Bernard et al. have calculated the photon energy spectrum and photon polarization in a heavy baryon chiral perturbation theory (HBχPT) including explicit Δ degrees of freedom [3]. Feynman diagrams of the various processes involved in the Bernard et al. calculation are shown in Fig. 1. It should be noted that the Gaponov and Khafizov calculation, performed in a purely QED framework, takes into account only proton and electron bremsstrahlung. The HBχPT calculation, however, includes all terms to order 1/*M* (*M* being the nucleon mass) including photon emission from the effective weak vertex. Interestingly, these additional terms contribute only at the < 0.5 % level and create only a very slight change in the final photon spectrum and branching ratio calculations; both the photon energy spectrum and the photon polarization observables are therefore dominated by electron bremsstrahlung. Calculations of the branching ratio from both groups are plotted in Fig. 2. The HBχPT prediction from Bernard et al. and the QED prediction of Gaponov and Khafizov are virtually indistinguishable.

These predictions show that the radiative decay mode of the neutron, while a rare process, should be observable in the laboratory. Indeed, a recent experiment attempting to measure this branching ratio has been performed by Beck et al. at Institut Laue-Langevin (ILL) in Grenoble, France. Due to experimental difficulties that produced very large backgrounds, this decay mode was not seen, and the result published was an upper limit of Br < 6.9 × 10^−3^ (at the 90 % confidence level) for detection of photons between 35 keV and 100 keV [4]. This result is compatible with the theoretical prediction of 2.21 × 10^−3^ for the same energy window [3].

## 2. Experimental Concept

**Fig. 3:**
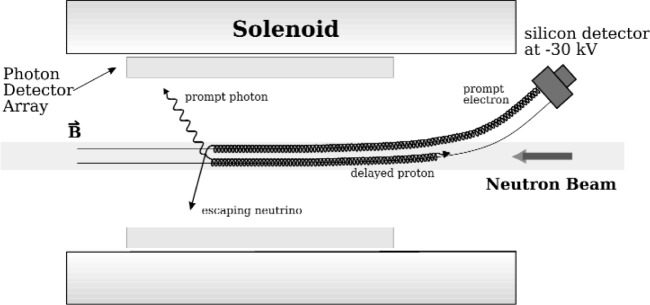
The NIST radiative neutron beta-decay setup. An electrostatic mirror (not shown) will redirect protons in the beam direction back into the charged particle detector.

We have designed an experiment to maximize the solid angle for detection of all decay products (apart from the antineutrino) and to reduce the probability of correlated background events. It uses the existing proton trap apparatus [5] which was used to make an in-beam measurement of the neutron lifetime [6]. A diagram of the experiment, mounted on the NG-6 endstation at the NCNR, is shown in Fig. 3. A cold neutron beam enters a homogeneous 4.6 T magnetic field produced by a superconducting solenoid. As the neutrons in the beam decay, the charged decay products are confined into tight cyclotron orbits moving along the magnetic field lines. The solenoid has been designed to have a slight (9.5°) bend in the magnetic field direction at one end allowing the decay proton and electron to be guided out of the beam and into a charged particle detector held at a high negative potential (≈ −30 kV) to accelerate the low energy protons to detectable energies. The electron, with a much higher energy (several hundred keV), reaches the charged particle detector first, and then the much slower proton (maximum energy of 751 eV) will drift to the detector a few microseconds later. A electrostatic mirror (not shown) will be used to re-direct protons with initial momenta in the beam direction back into the detector. The magnetic field and electrostatic mirror provide for over 2π solid-angle coverage for detection of the charged decay products.

For a radiative decay, the photon is detected by an concentric array of photon detectors around the neutron beam in the bore of the solenoid. Only triple electron-proton-photon coincidences will be counted as radiative decay events. The delayed coincidence between proton and electron detection provides a powerful method for rejecting background photons that are not associated with a neutron decay. The placement of the charged particle detector well away from the photon detection region also reduces the correlated background from bremsstrahlung photons from the decay electron slowing in the charged particle detector.

## 3. Development

**Fig. 4:**
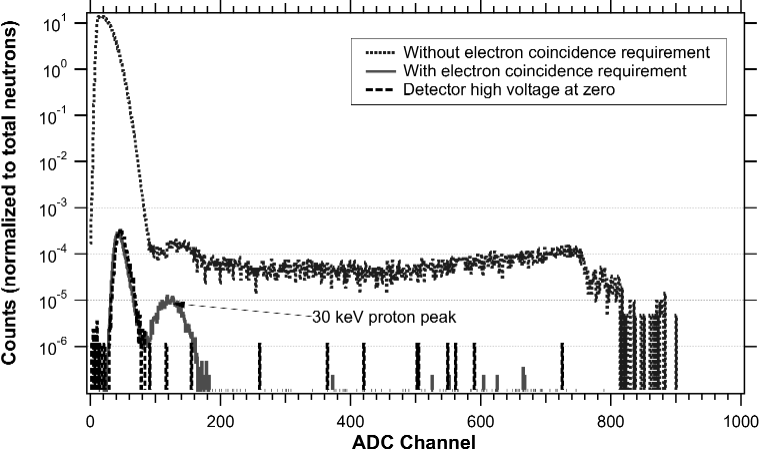
Pulse-height spectra from the electron-proton silicon surface barrier detector. Shown are the singles spectrum (dotted line at top), the coincidence spectrum (showing the energy spectrum of protons detected in delayed coincidence with a prompt electron) with the −30 kV detector high-voltage on (solid line), and the coincidence spectrum with the detector high-voltage off. (dashed line.)

Initial measurements of the electron-proton coincidence rate while the proton-trap lifetime apparatus was last installed on NG-6 showed a total rate of 0.2 s^−1^ with low backgrounds. This measurement was performed with a roughly 1 cm diameter cold neutron beam; the pulse-height spectrum that resulted is shown in Fig. 4. This shows good separation of protons in coincidence with electrons, with little background under the proton peak. With several geometric and detector optimizations (including a larger diameter beam, giving a greater rate), this rate should be improved by a factor of 100.

**Fig. 5:**
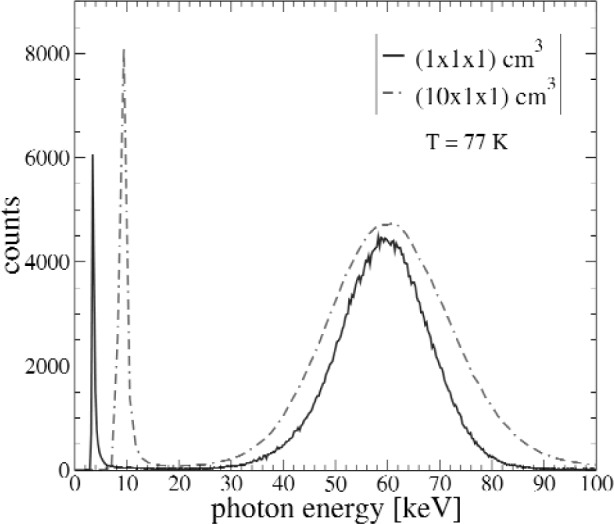
Spectra of gamma-rays from a ^241^Am source. Shown are spectra for both (1 × 1 × 1) cm^3^ and (1 × 1 × 10) cm^3^ BGO crystals, with a 1.3 cm × 1.3 cm APD coupled to one end-face on each.

The major challenge in this experiment is the photon detector array development. We are currently investigating bismuth germanate (BGO) and pure CsI scintillation detectors coupled to silicon avalanche photodiodes (APDs), each of which can operate in the low-temperature, high magnetic field environment inside the magnet. Since a lower photon energy threshold implies a larger signal, optimizing for low-energy photon detection is key. Both BGO and pure CsI (as well as most un-doped inorganic crystals) exhibit increased light output at low temperatures, which allows for improved low-energy thresholds. For example, the light output of BGO is approximately 2.5 times greater at 77 K than at room temperature [7]. Similarly, the gain of the APD increases and its noise decreases as the temperature decreases [8]. Gamma-ray energy spectra from an ^241^Am source taken with BGO crystals are shown in Fig. 5. As can be seen, the energy threshold for the 10 cm long crystal is ≈ 15 keV, up from ≈ 7 keV for the 1 cm crystal. This increase can be attributed to the decreased light collection in the longer crystal.

Since we wish to measure the photon spectrum, a detailed model of the response function of the detector array is needed. An effort to fully model the photon detection region with Monte Carlo techniques is in progress.

## 4. Current Status and Future Work

The apparatus has been installed and aligned at the fundamental physics station NG-6 at the NCNR. Optimization tests of the electron-proton coincidence setup are currently in progress using the cold neutron beam. We are investigating the long-term gain stability of the APD plus scintillation crystal system and finalizing the design of the photon detector array. Our initial design idea is to line the inside of the solenoid bore with twelve 20 cm long scintillation crystals with APD readout. The array would be thermally sunk to the 77 K liquid nitrogen shield of the solenoid providing the necessary cooling to obtain the increased light output and gain.

Preliminary count-rate estimates predict a triple-coincidence (electron-proton-photon) rate of 0.02 s^−1^. Given a uncorrelated gamma-ray background rate above 30 keV of about 500 s^−1^ (giving a false coincidence rate of 0.002 s^−1^), the signal-to-background ratio is still 10:1. Judicious placement and shielding of the photon detectors will keep this background to reasonable levels. If the signal-to-background ratio is kept at 10:1, after a single reactor cycle (roughly 30 days) not only will this decay mode be definitively seen for the first time, but the spectrum of photons from radiative *β*-decays will be measured at the 5 % level.

If this technique for detection of radiative neutron beta decay proves fruitful, a more precise measurement of the photon spectrum should be possible. A measurement at the < 1 % level could reveal the contributions from weak vertex and provide an alternative determination of the weak coupling constants *g_V_* and *g_A_*. Similarly, if the photon’s circular polarization could be measured, it would reveal information about the Dirac structure of the weak current [3].

## 5. Conclusion

Neutron radiative beta-decay has never been definitively observed. We intend to measure the photon spectrum making use of an existing apparatus that will give us a high electron-proton coincidence rate with a low photon background. Efficient detection of photons above ≈ 10 keV will be provided by inorganic scintillation crystals coupled to avalanche photodiodes. We hope to not only definitively see radiative neutron beta-decay for the first time, but perform a 5 % measurement of the photon spectrum. We hope to be taking data by late 2004.
